# Use of Three-Dimensional Echocardiography to Identify an Unusual Cause of Aortic Regurgitation

**DOI:** 10.1016/j.case.2024.03.002

**Published:** 2024-04-11

**Authors:** J. Kyle Buck, Manrique Alvarez, Sneha Chebrolu, Rohesh J. Fernando, Karl Richardson, Adrian L. Lata, Scott R. Coleman

**Affiliations:** a Department of Anesthesiology, Wake Forest Baptist at Atrium Health, Winston-Salem, North Carolina; b Department of Cardiology, Wake Forest Baptist at Atrium Health, Winston-Salem, North Carolina; c Department of Cardiothoracic Surgery, Wake Forest Baptist at Atrium Health, Winston-Salem, North Carolina

**Keywords:** Aortic valve, Aortic regurgitation, Coronary angiography, Percutaneous coronary interventions, 3D transesophageal echocardiography

## Abstract

• New-onset AR after coronary angiography should raise suspicion for AV injury.
• Consider 3D imaging if no clear etiology found with 2D imaging.
• Three-dimensional imaging showed a partial tear of RCC.
• Medical intervention was altered based in part on TEE findings.

## Graphical abstract

**Figure:**
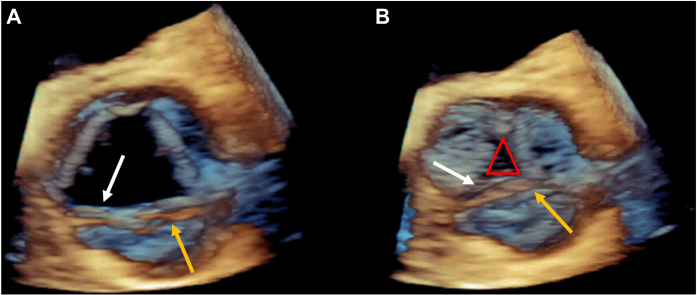

## Introduction

Aortic valve (AV) injury during invasive coronary angiography (ICA) is uncommon. The reported incidence of this complication is 0.008% to 0.02% during diagnostic catheterization and 0.06% to 0.07% during percutaneous coronary intervention (PCI).^1^ Often, the injury is noted quickly due to acute-onset aortic regurgitation (AR).^2^ This report presents a case of iatrogenic injury of the AV resulting in insidious AR. Injury to the right coronary cusp (RCC) was identified via intraoperative transesophageal echocardiography (TEE). Three-dimensional imaging was utilized to delineate this from other pathologies such as endocarditis, fibroelastoma, and Lambl’s excrescence.

## Case Presentation

The patient was a 63-year-old woman with medical history significant for hypertension, type 2 diabetes mellitus, obesity (body mass index, 44 kg/m^2^), rheumatoid arthritis treated with methotrexate, and coronary artery disease with prior percutaneous drug eluting stent placement to the circumflex artery. The patient was evaluated at an outside hospital for progressive, intermittent substernal chest pain. Unstable angina was diagnosed, and a drug eluting stent was placed to correct a culprit lesion identified in the right coronary artery. Transthoracic echocardiography (TTE) performed approximately 12 hours prior to ICA demonstrated normal biventricular systolic function with no significant valvular abnormalities. The AV was difficult to visualize, but color-flow Doppler showed no evidence of AR (Videos 1 and 2). The postprocedural course was uneventful, and the patient was discharged home the next day. Two days after discharge, they began to experience worsening shortness of breath. The patient was diagnosed with acute hypoxic respiratory failure thought to be secondary to heart failure exacerbation, with chest x-ray demonstrating bilateral pulmonary edema, and an elevated brain natriuretic peptide. The patient was treated with diuretics and discharged home after 1 week. No repeat echocardiogram was performed during this visit.

**Figure 1:**
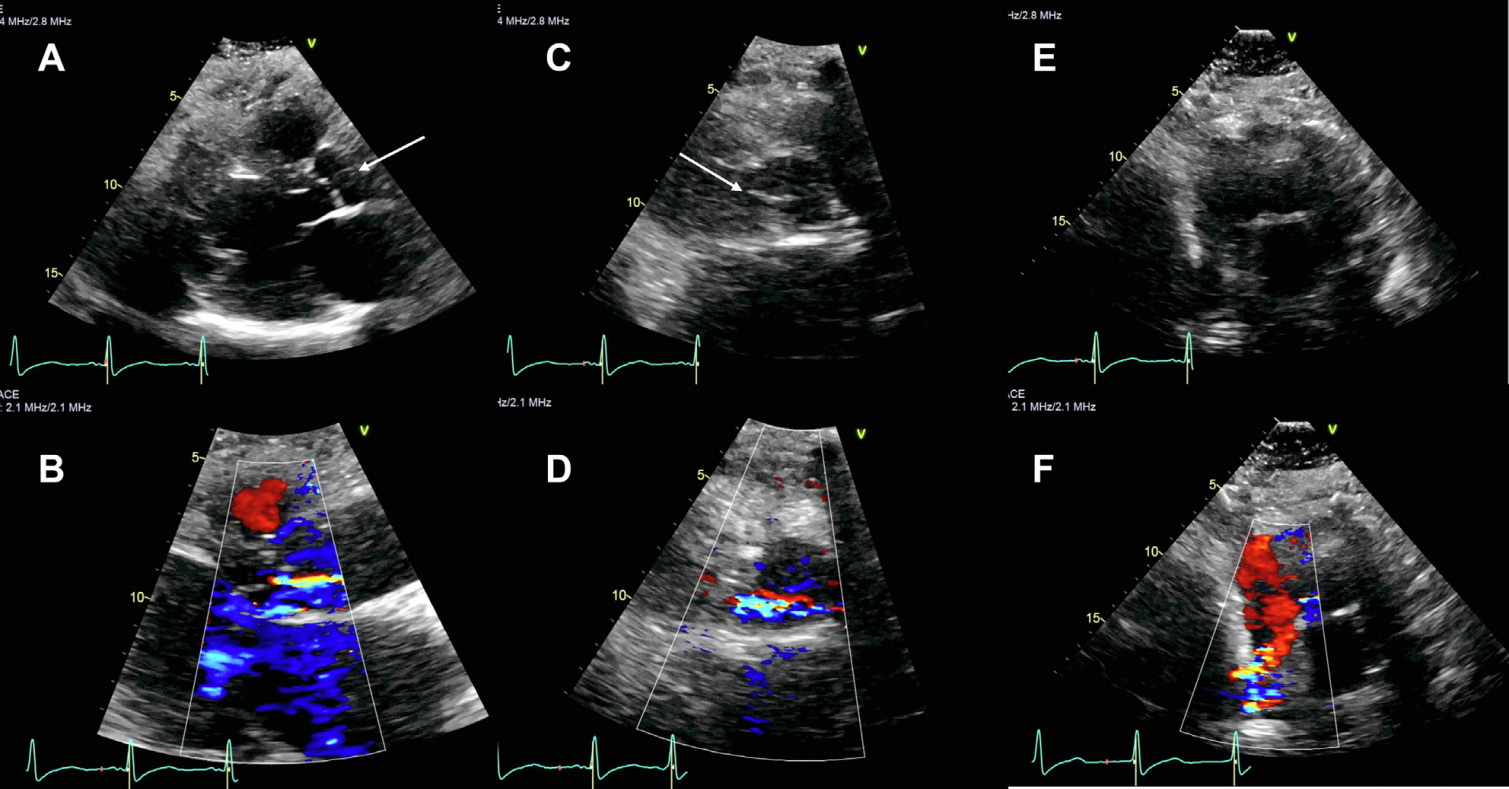
Two-dimensional TTE (performed 13 days after ICA), diastolic images without (*top row*) and with (*bottom row*) color-flow Doppler, demonstrated an unexplained echodensity (*arrows*) between the noncoronary cusp and RCC that appeared to be attached to the AV, best seen in the parasternal long axis **(A)** and parasternal short-axis **(C)** views. The associated AR in these views was reported as mild **(B, D)**, originated between the noncoronary cusp and RCC, and was eccentrically directed toward the RCC. Apical views were suboptimal **(E)**, but mild eccentric AR was noted **(F)**.

**Figure 2:**
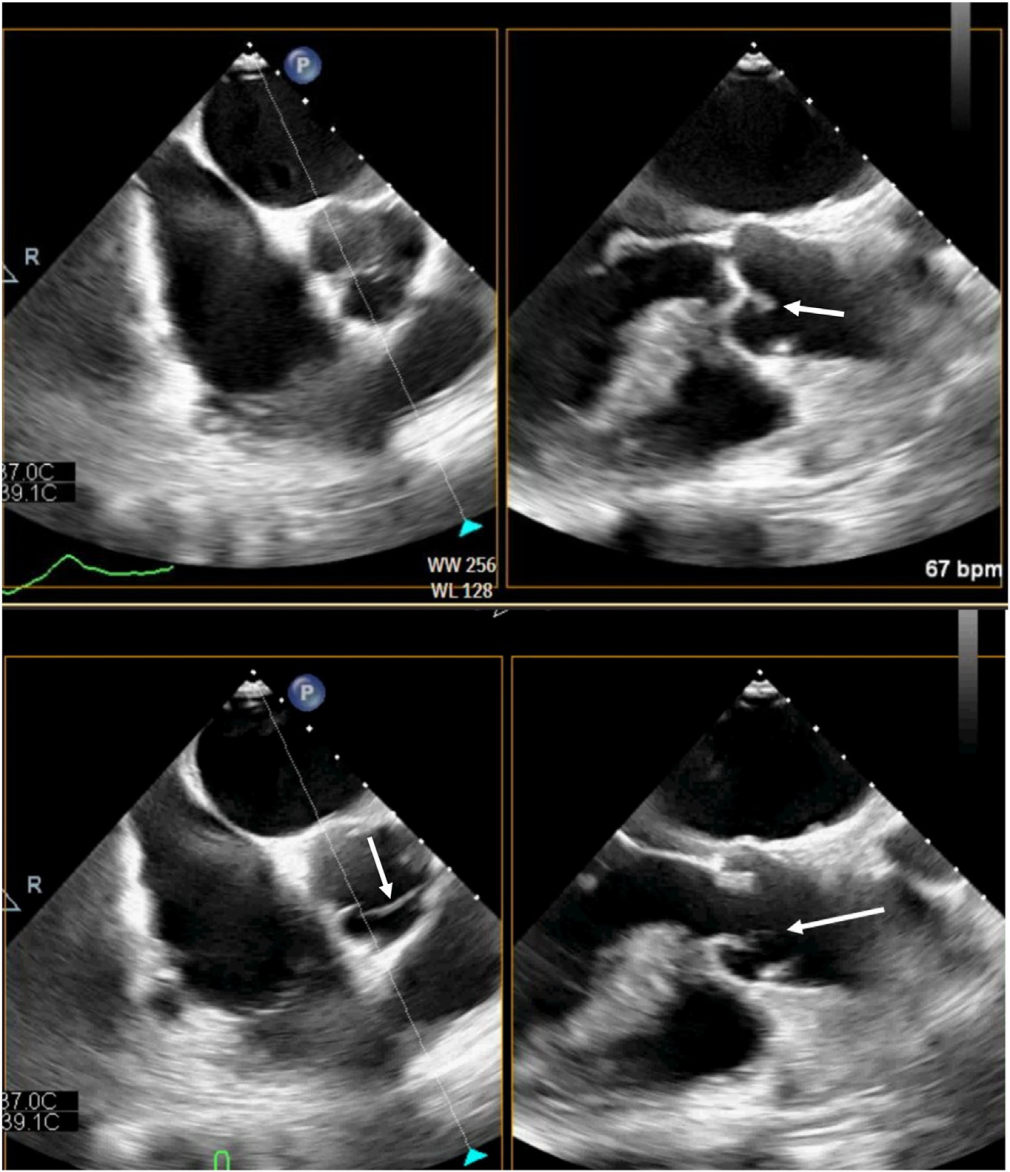
Two-dimensional TEE (preoperative), midesophageal AV biplane views, short-axis (*left*) and long-axis (*right*), diastolic (*top*) and systolic (*bottom*) views, demonstrates an echodensity on the aortic side of the valve suspicious for Lambl’s excresence, distal leaflet tissue, or artifact (*arrows*). This was later demonstrated at surgery to represent partial detachment of the distal portion of the RCC.

Ten days after coronary stent placement, the patient presented to the emergency department complaining of dyspnea, orthopnea, and worsening bilateral lower extremity edema, as well as intermittent fever and productive cough. The patient reported measuring an oxygen saturation of 75% on a home pulse oximeter. Chest x-ray performed in the emergency department demonstrated diffuse prominent pulmonary vasculature. The patient was admitted to the cardiology service, and treatment was initiated for heart failure exacerbation with intravenous diuretics, as well as treatment for potential hospital-acquired pneumonia. The patient was noted to have a diastolic blood pressure near 50 mm Hg, which was a decrease from 70 to 80 mm Hg prior to the PCI along with a high-pitched diastolic murmur at the right upper sternal border. These findings prompted a repeat TTE. New eccentric AR was seen with a pressure half time of 350 ms (Figure 1, and Videos 3 and 4). The AR was described as moderate but was likely underestimated due to the eccentricity and inability to fully visualize the regurgitant jet on TTE. To further evaluate the patient for endocarditis, a TEE was performed. This showed moderate-to-severe AR by visual estimation along with a thin, mobile structure on the aortic side of the AV, initially thought to be a Lambl’s excrescence (Figure 2, Video 5). However, there was also concern that this mass could represent an infectious vegetation given the infectious symptoms and recent ICA.

**Figure 3:**
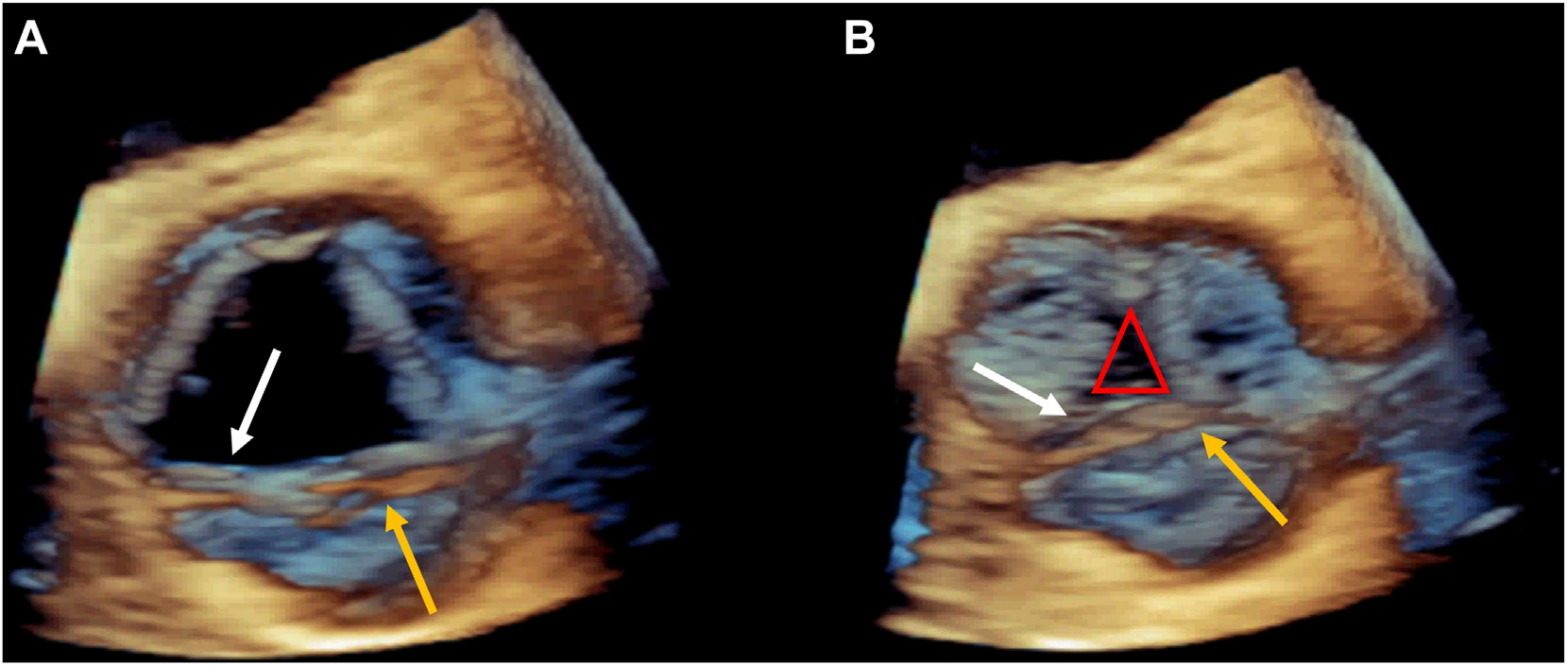
Intraoperative 3D volume-rendered TEE images of the AV, zoomed short-axis display from the perspective of the aortic root in systole **(A)** and diastole **(B)**, demonstrate a flap of tissue (*yellow arrow*) distal to the RCC (*white arrow*) that appears to be tethered at the commissures and a central coaptation defect (*red triangle*).

Cardiothoracic surgery was consulted for AV replacement. Given negative blood cultures and increasing suspicion for iatrogenic AR rather than endocarditis, the patient was deemed an acceptable candidate for surgical AV replacement and brought to the operating room where intraoperative TEE was performed. Two-dimensional (2D) TEE imaging showed moderate AR and a structure resembling a Lambl's excrescence (Video 6). There was no flow reversal in the descending thoracic aorta, and the three-dimensional (3D) effective regurgitant orifice area was 0.11 cm^2^. During further inspection of the AV, the pathology appeared to be favoring the RCC and the mobile echodensity was more consistent with loose valve tissue rather than a Lambl's excrescence (Video 7). Three-dimensional imaging was obtained to further assess the AV and demonstrated what appeared to be a flap originating from the distal edge of the RCC (Figure 3, Video 8). The 3D appearance suggested a portion of the distal edge of the patient's RCC had separated from the remainder of the leaflet. Because of this, the RCC was not long enough to coapt with the other 2 cusps.

**Figure 4:**
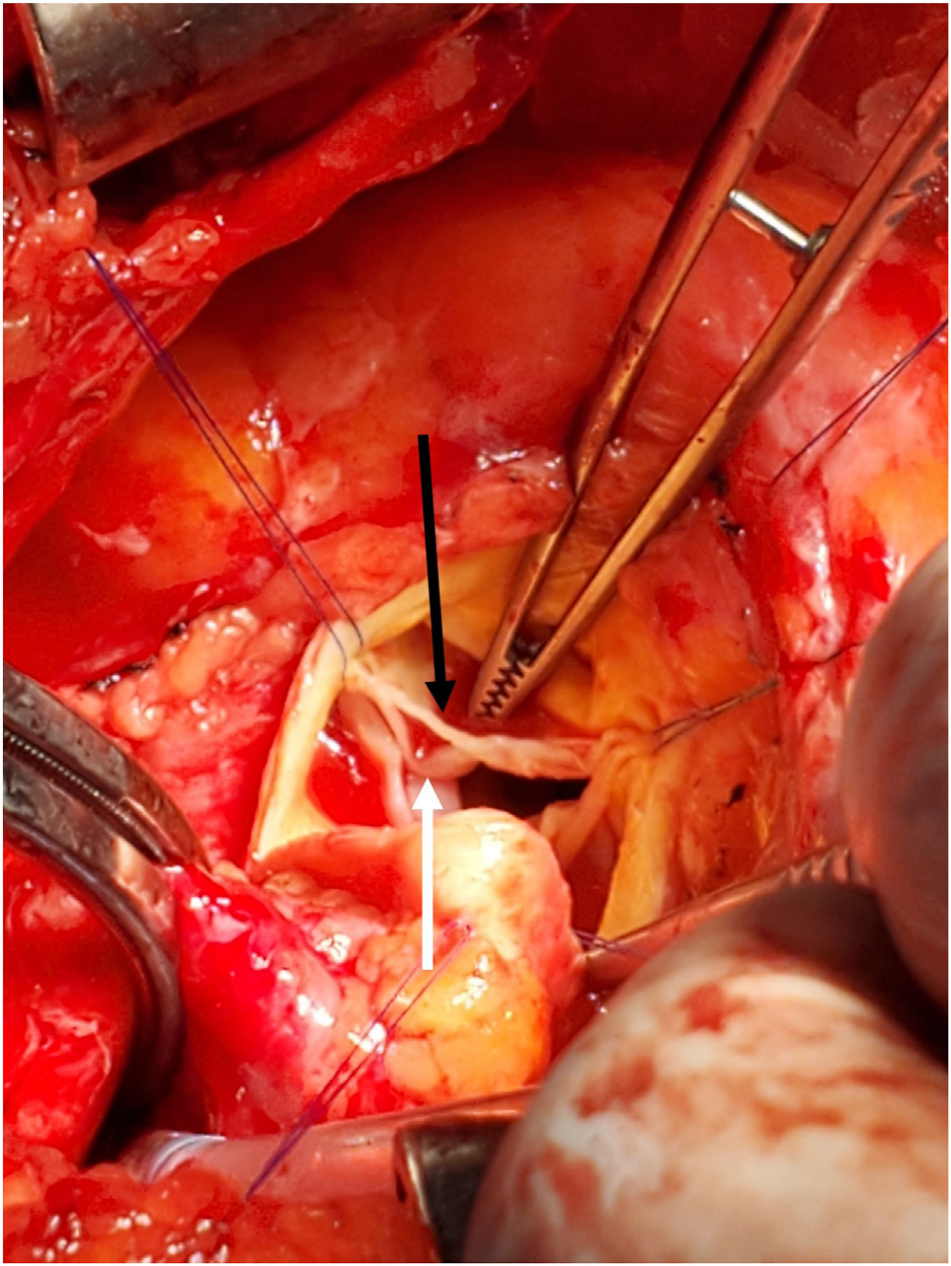
Intraoperative surgical photograph of the exposed AV demonstrates partial detachment (*black arrow*) of the distal portion of the RCC (*white arrow*).

Surgical AV replacement was completed via median sternotomy. Exploration of the native AV demonstrated that a band of tissue on the edge of the RCC had indeed separated from the remainder of the leaflet (Figure 4), presumably torn by a coronary catheter during the recent PCI. The patient recovered uneventfully, and postoperative echocardiography demonstrated a well-functioning prosthetic AV with no residual AR. As the intraoperative findings allayed concerns about infective endocarditis, the patient did not require an extended antibiotic course beyond the normal perioperative prophylaxis. They were discharged on postoperative day 6 and have had no further known complications.

## Discussion

The patient's symptoms along with widened pulse pressure and diastolic murmur after ICA raised suspicion for valvular injury. A TTE was obtained to evaluate the AV as well as search for other causes of heart failure. Cardiac computed tomography or cardiovascular magnetic resonance were not performed but can provide complementary diagnostic options to evaluate the etiology and severity of the AR, respectively. Eccentric AR was the primary finding, and TEE was performed given concerns that eccentric AR can be underappreciated and therefore underestimated on TTE. In addition to the AR, the TEE identified a mobile structure consistent with a Lambl's excrescence (Video 8). A Lambl's excrescence is generally described as a filamentous, hypermobile structure originating at the coaptation point of a valve, measuring approximately 1 mm in diameter while extending up to 10 mm in length.^3^ They are postulated to occur due to papillary outgrowth, hyalinization, and fibrosis at highly stressed valve closure lines.^3^ However, these structures are almost always incidental discoveries with unclear clinical significance.

Aortic regurgitation occurs due to either valvular disease or alteration of aortic root anatomy.^4^ The first objective was to rule out aortic pathology. With the recent PCI and no prior indication of aortic root dilation, dissection of the aorta was more likely. Inspection of the proximal aorta yielded no signs of dissection or dilation, and we transitioned to examining the AV for pathology.

Upon early inspection, an echodensity on the aortic side of the AV was identified. The differential diagnosis for masses on the AV includes neoplasm (such as papillary fibroelastoma or myxoma), calcification, thrombosis, bacterial vegetation or abscess, and Lambl's excrescence.^5^ Based on the history, AV vegetation and iatrogenic injury to the AV appeared to be more likely. Neoplasia seemed less likely given the absence of valve pathology on imaging only weeks prior. Papillary fibroelastoma is the neoplasm that most commonly affects the intracardiac valves, accounting for 45% of all valvular tumors in one study.^5^ Although these tumors are histologically benign, they can result in stroke, valvular dysfunction, or even sudden cardiac death secondary to occlusion of the coronary ostia.^6^ Echocardiographically, a papillary fibroelastoma generally appears pedunculated and mobile, with homogeneous speckling.^3^ Comparing the series of echocardiograms showed an acute change in the valve morphology and the AR. The acute nature of these findings helped narrow the differential diagnosis to an acute process such as endocarditis or iatrogenic injury.

The patient's report of fever occurring since PCI in conjunction with the mobile echodensity raised the possibility of infective endocarditis. Reassuringly, the patient had no other identifiable valvular lesions and no evidence of bacteremia based on blood cultures. No infectious source was identified during the hospital course to explain the fever. Perhaps the pulmonary edema that developed from the acute AR caused the fever.^7^ Additionally, the structure in question did not have the typical characteristics of irregular shape and heterogeneous composition consistent with a vegetation.^8^

After a complete 2D evaluation of the AV, the cause of AR was not yet clear so 3D imaging was employed to elucidate the cause of the AR. The 3D imaging showed the mobile lesion was not a single strand arising from a coaptation line but instead a band of tissue arising from the annular edges of the RCC (Video 7).

Three-dimensional echocardiography continues to become more accessible, and as technology improves, use of this modality in the fast-paced environment of the operating room has become more widespread. Three-dimensional assessment of the AV has been described as superior to 2D imaging. Assessment of aortic masses and AR is improved with 3D imaging.^4^ The use of 3D imaging provided us with a diagnosis for the patient. Importantly, this identification helped to avoid prolonged antibiotic treatment as it was no longer deemed necessary.

Surgical exploration confirmed that the coapting edge of the RCC of the AV had sheared from the remaining leaflet tissue, causing both the AR and the sudden appearance of a mobile structure arising from the AV. As these findings were not present prior to ICA, it is likely that valve injury occurred during that procedure. Although this is a rare complication, there are prior case reports documented.^1^^,^^2^^,^^9^^,^^10^

The eccentricity of the AR jet likely affected the physical exam and ability to accurately identify and quantify the AR severity. The jet was anteriorly directed, making it easier to auscultate on physical exam, which is the finding that prompted repeat echocardiography. However, the eccentricity reduced the sensitivity of TTE in assessing the AR and accurately grading the severity. Transesophageal echocardiographic imaging will have better sensitivity, but measurements of severity, such as vena contracta and jet—to–left ventricular outflow tract width ratio, are often underestimated with eccentric AR. Three-dimensional imaging should provide improved assessment compared with 2D evaluation.

A few factors likely delayed the diagnosis for this patient. First, the coronary artery disease and recent intervention made it reasonable to think the symptoms were related to heart failure. Also, transfer in care between facilities was a barrier. The procedure report and fluoroscopy from the PCI did not describe any difficulty. Retrospectively, it was learned from the cardiologist that the procedure was challenging and required several catheter exchanges. Transthoracic echocardiographic imaging and other documentation from the outside institution also were not available upon the patient's readmission. This process took days, and it was the physical exam findings that led to the initial suspicion of AR as the cause of the heart failure symptoms. Based on the patient's history and symptoms, the AR was likely severe throughout the hospital course. A higher index of suspicion for valvular injury and thorough cardiac auscultation would have likely led to quicker echocardiography and a quicker diagnosis.

## Conclusion

This case demonstrated an unusual mechanism for acute AR secondary to apparent shearing of an AV leaflet by a coronary catheter. The case also reinforces the high level of suspicion providers need to have in the case of clinical symptoms of heart failure and new AR. The limitations of 2D echocardiography for eccentric AR are illustrated, while 3D imaging of the AV by TEE revealed a more complete impression of the abnormality. Elucidating the etiology of the valvular lesion allowed for optimizing treatment for the patient and avoided unnecessary long-term antibiotic therapy. Iatrogenic injury to the AV, while rare, should be considered in patients presenting with acute signs and symptoms of heart failure after ICA.

## Ethics Statement

The authors declare that the work described has been carried out in accordance with The Code of Ethics of the World Medical Association (Declaration of Helsinki) for experiments involving humans.

## Consent Statement

Complete written informed consent was obtained from the patient (or appropriate parent, guardian, or power of attorney) for the publication of this study and accompanying images.

## Funding Statement

The authors declare that this report did not receive any specific grant from funding agencies in the public, commercial, or not-for-profit sectors.

## Disclosure Statement

The authors report no conflict of interest.

## Supplementary Data

Video 1 Two-dimensional TTE (performed 12 hours prior to ICA), apical 5-chamber narrow sector view of the AV, demonstrates apparently normal leaflet motion with no evidence of a mass.

Video 2 Two-dimensional TTE (performed 12 hours prior to ICA) with color-flow Doppler, apical 5-chamber narrow sector view of the AV, demonstrates no evidence of aortic stenosis or regurgitation.

Video 3 Two-dimensional TTE (performed 13 days after ICA), apical 5-chamber view of the AV, demonstrates a previously unseen band of tissue distal to the AV near the RCC.

Video 4 Two-dimensional TTE (performed 13 days after ICA) with color-flow Doppler, apical 5-chamber view of the AV, demonstrates a new eccentric AR jet directed toward the anterior mitral valve leaflet.

Video 5 Two-dimensional TEE, midesophageal biplane AV view and short-axis (*left*) and orthogonal long-axis (*right*) views, demonstrates an unexplained echodensity distal to the AV and near to and possibly connecting the RCC to the sinotubular junction.

Video 6 Intraoperative 2D TEE, midesophageal AV long-axis (136°) view with color-flow Doppler, demonstrates moderate AR with a central origin and eccentric, anteriorly directed jet. An echodensity is also noted on the tip of the RCC.

Video 7 Intraoperative 2D TEE, midesophageal AV long-axis (133°) view, demonstrates an unexplained mobile mass attached to the RCC of the AV, later demonstrated at surgery to represent partial detachment of the distal portion of the RCC.

Video 8 Intraoperative 3D volume-rendered TEE images of the AV, zoomed short-axis display from the perspective of the aortic root, demonstrate a flap of tissue distal to the RCC that appears to be tethered at the commissures and a central coaptation defect.
